# Ultrasound beam steering of oxygen nanobubbles for enhanced bladder cancer therapy

**DOI:** 10.1038/s41598-018-20363-8

**Published:** 2018-02-15

**Authors:** Pushpak Bhandari, Gloriia Novikova, Craig J. Goergen, Joseph Irudayaraj

**Affiliations:** 1 Department of Agricultural and Biological Engineering, Purdue University, West Lafayette, Indiana, 47907 United States; 2 Davidson School of Chemical Engineering, Purdue University, West Lafayette, Indiana, 47907 United States; 3 Weldon School of Biomedical Engineering, Purdue University, West Lafayette, Indiana, 47907 United States; 4 Purdue University Center for Cancer Research, West Lafayette, Indiana, 47907 United States; 5 Department of Bioengineering, UIUC, Urbana, IL 61801 United States

## Abstract

New intravesical treatment approaches for bladder cancer are needed as currently approved treatments show several side effects and high tumor recurrence rate. Our study used MB49 murine urothelial carcinoma model to evaluate oxygen encapsulated cellulosic nanobubbles as a novel agent for imaging and ultrasound guided drug delivery. In this study, we show that oxygen nanobubbles (ONB) can be propelled (up to 40 mm/s) and precisely guided *in vivo* to the tumor by an ultrasound beam. Nanobubble velocity can be controlled by altering the power of the ultrasound Doppler beam, while nanobubble direction can be adjusted to different desired angles by altering the angle of the beam. Precise ultrasound beam steering of oxygen nanobubbles was shown to enhance the efficacy of mitomycin-C, resulting in significantly lower tumor progression rates while using a 50% lower concentration of chemotherapeutic drug. Further, dark field imaging was utilized to visualize and quantify the ONB *ex vivo*. ONBs were found to localize up to 500 µm inside the tumor using beam steering. These results demonstrate the potential of an oxygen nanobubble drug encapsulated system to become a promising strategy for targeted drug delivery because of its multimodal (imaging and oxygen delivery) and multifunctional (targeting and hypoxia programming) properties.

## Introduction

Bladder cancer is the fourth most common cancer in men and tenth most common in women. In the U.S., nearly 74,000 new cases of bladder cancer were diagnosed in 2015^1^. Approximately 70% of patients with early stage non-muscle invasive bladder cancer (NMIBC) suffer disease recurrence after initial surgical treatment^2^. Hypoxia has been shown to significantly correlate with an unfavorable prognosis in bladder cancer^3–6^. Similarly, the efficacy of radiation therapy^7–9^ and chemotherapy^3,6,10^ can be significantly hampered by hypoxia in bladder cancer^11,12^. The contribution of hypoxia to chemoresistance, radioresistance, alteration of vasculature, chaotic blood flow, and genomic instability are well documented^13–17^. Normoxic malignant cells are 2 to 3 times more sensitive to cell death from radiation and chemo therapy than hypoxic malignant cells^18^. It is well known that targeting hypoxia enhances the efficacy of chemotherapy and radiation therapy^19,20^.

While image guided-methods based on MRI exist for guided drug delivery, these require expensive and extensive instrumentation^21,22^. We hypothesize that an ultrasound (US) beam mediated approach to steer oxygen nanobubbles (ONB) to the tumor site will not only increase the localization of ONBs at the tumor, but will also aid in oxygenation and possible penetration of the bubbles into the tumor vasculature based on sonoporation. We also expect oxygenation will sensitize hypoxic cells to subsequent chemotherapy because normal cells are two to three times more receptive to subsequent therapies compared to hypoxic cells^11,12,20^.

The adjuvant chemotherapy of choice for NMIBC is intravesical instillation of bacillus Calmette-Guérin (BCG) or mitomycin-C (MMC)^23^ While mitomycin-C delays disease progression of NMIBC in most patients, long-term follow-up reveals a high recurrence rate (approximately 30–70% in over 10 years)^23,24^. Retreatment with BCG or MMC can be efficacious, but often is limited by increased localized or systemic toxicity, leading to cessation of treatment^23,25^. Furthermore, the development of resistance to BCG therapy is common^26^. Treatment of advanced stage, muscle invasive cancers localized to the bladder with BCG is not effective, thus cystectomy is often performed, which dramatically impacts patient’s quality of life.

**Figure 1:**
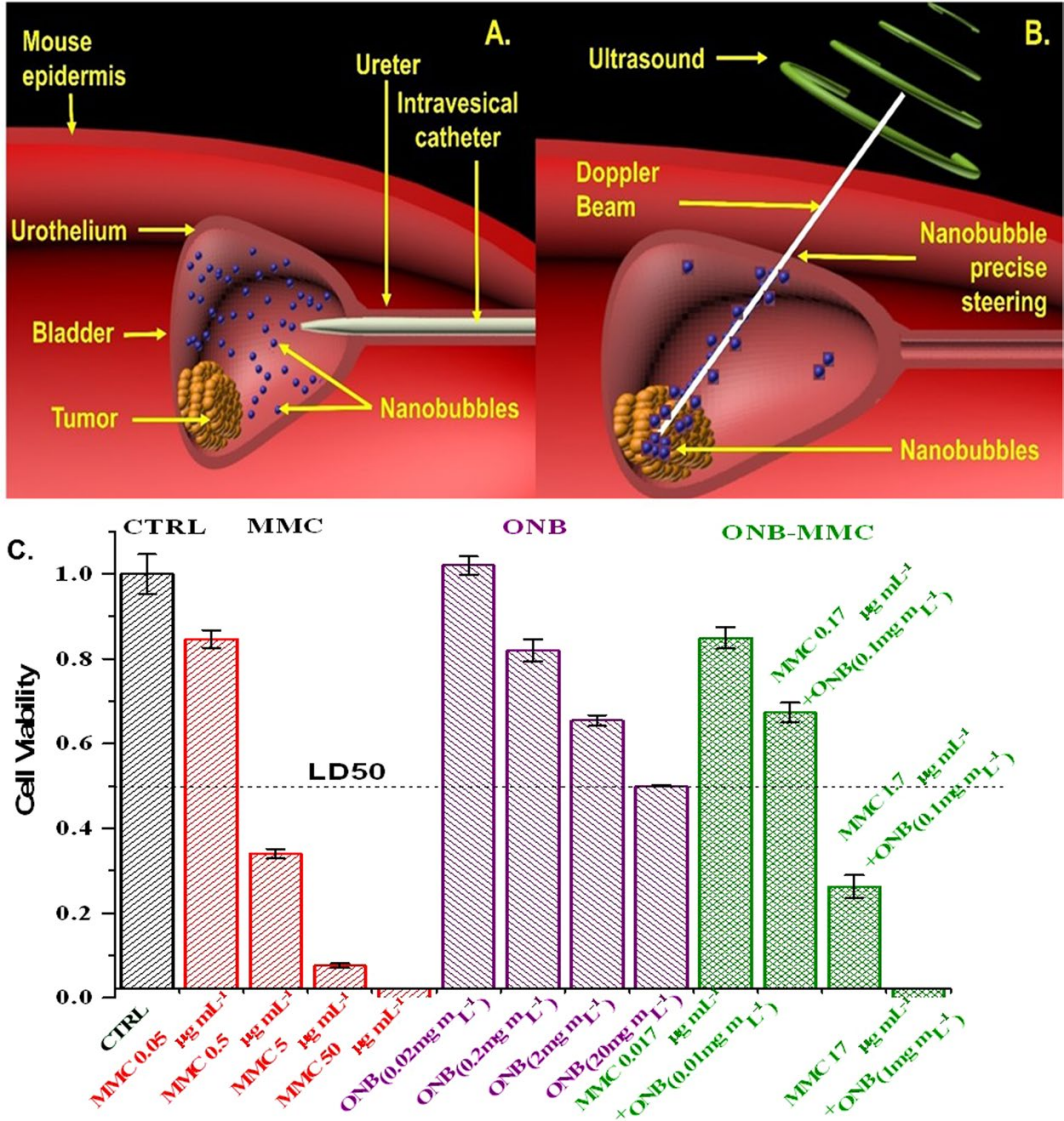
Schematic of intravesical treatment in mice bladder cancer. (**A**) Therapy introduced into the bladder by intravesical catheter. (**B**) Precise localization of mitomycin-C loaded ONBs (in blue color) to the hypoxic tumor microenvironment using Doppler ultrasound steering with simultaneous B-mode US imaging. (Not to scale). (**C**) Oxygen nanobubbles can halve the dose of MMC required with enhanced therapeutic efficacy, *in vitro*. Cell viability was assessed with MTT assay for different conditions of CTRL, MMC, ONB, and ONB-MMC.

In this study, we show that nanosize cellulosic oxygen bubbles can be used for drug encapsulation and precise drug delivery by propelling the nanobubbles using an ultrasound Doppler beam. Re-oxygenation of the hypoxic tissue was shown to destabilize hypoxia driven pathways and significantly suppress tumor progression^27,28^. Here, we show that ONBs can be propelled along the ultrasound Doppler beam while being imaged in real time *in vivo*. For intravesical therapy, first, the nanobubbles (NBs) were injected via a catheter in the bladder of a mouse (Fig. 1A). Upon confirmation of ONB localization using B-mode ultrasound, pulsed wave Doppler ultrasound was employed to precisely steer the ONBs to the hypoxic regions of the tumor (Fig. 1B). Precise drug delivery by means of beam steering allows us to guide the nanobubbles directly onto hypoxic tumor region, thus, making the nanobubbles permeate the tumor tissue and efficiently deliver the desired drug. In this study, we show that the amount of chemotherapeutic drug can be decreased by 50% and significantly lower tumor progression rates can be achieved. Consequently, our unconventional approach provides an injectable, nanoscale delivery platform that significantly enhances the efficacy of chemotherapeutic agents by precise drug delivery and targeted re-oxygenation of hypoxic tumor regions.

## Results and Discussion

### Synthesis and Optimization of ONB Efficacy *In Vitro*

Oxygen nanobubbles (ONBs) with a size of 200 nm were synthesized using crosslinking chemistry to encapsulate oxygen within a sodium carboxymethylcellulose shell^27,29^. First, cell cytotoxicity assays were carried out with MB49 cell line to evaluate the effect of ONB (0.02–20 mg/mL) and MMC (0.05–50 µg/mL) (Fig. 1C). We found that both treatments had a significant effect on cell viability compared to control (no treatment). MMC had the most pronounced effect on cell viability with 0.5 µg/mL MMC concentration resulting in ~30% cell viability. ONB’s were significant in reducing cell viability to ~50% at the highest synthesized concentration of 20 mg/mL. Oxygen concentrations in the culture media increased significantly upon addition of ONBs indicating that the therapeutic effect could be due to perturbation of the hypoxia-adaptive pathways^27^.

Next, we hypothesized that the oxygenation due to ONBs can synergistically increase the effectiveness of MMC. We prepared MMC-ONB drug combinations by crosslinking MMC within the ONB shell (See Methods) and observed the viability of MB49 cells. It was found that a dose consisting of 17 µg/ml MMC and 1 mg/mL ONB yielded complete cell death (Fig. 1C). The ONB dose of 1 mg/mL contributed to halving of the dose of MMC while still yielding complete cell death. Thus, we found that the simultaneous effect of MMC and ONB resulted in a significant increase in cell death and can help reduce the concentration, and thus, the toxicity and side effects of MMC.

### *In Vitro* Beam Steering Optimization

The effect of beam steering on cell viability was studied using a factorial design of experiments and response surface methodology using MMC (0.05–5 µg/mL), ONB (0.2–20 mg/mL) and beam steering (ON/OFF) as variables (Supporting Information Table S1). Both, MMC and ONB were significant in decreasing cell viability and our results indicated that beam steering had a significant effect in decreasing cell viability when MMC (0.5 µg/mL) and ONB (2 mg/mL) were at ‘medium’ concentration (Figure S1). The ultrasound beam steering was only significant in increasing the efficiency of ONB-MMC combination therapy. No significant effect of the beam was observed on the MB49 cells treated individually with ONB or MMC. We hypothesize that oxygen nanobubbles can have an improved effect when they are uptaken by cells compared to only oxygenating the cellular microenvironment. Previously, we demonstrated the uptake of ONB without beam steering in prostate cancer cells using darkfield microscopy^29^. Thus, from *in vitro* experiments, we concluded that the combination of ONB-MMC was found to be highly significant in reducing the viability of MB49 bladder cancer cells and the effect of beam steering was found to have a pronounced effect on the combinatorial treatment, compared to the individual ONB or MMC treatments.

Further, we investigated the parameters influencing nanobubble velocity under the Doppler ultrasound beam by conducting a two-level factorial design (Supporting Information Table S2) with four parameters: bubble size (200 nm and 800 nm), ultrasound power (20% and 100%), ultrasound frequency (32 MHz and 40 MHz) and beam angle (0° and 15°). We found that both, beam power and beam frequency were significant in increasing the velocity of the nanobubble (*P* < 0.01). The beam angle did not appear to be significant in changing the velocity of the nanobubble.

To determine the oxygen release profile under an ultrasound beam, we measured the oxygen release in MB49 cells treated with saline (CTRL) or ONB using an OxyLite oxygen probe aseptically inserted into the cell culture (Figure S3)^27^. Briefly, no significant increase in oxygen concentration was observed using the ultrasound beam for either the ONB (P > 0.5) or the saline group (P > 0.5). The group treated with ONB had a significantly higher oxygen content without the beam compared to the saline group likely due to passive diffusion of oxygen from nanobubbles.

### *In Vivo* Characterization of Beam Steering

Beam steering experiments were performed *in vivo* in mice in the absence of tumors to investigate how the ultrasound beam power influences nanobubble velocity. The average velocity of ONB was observed to significantly increase as the power increased from 5% to 100%, making it possible to direct nanobubbles at desired velocities by tuning the power of the ultrasound Doppler beam (Figure S2). Using these results, we utilized 100% of the transducer’s power for future experiments to achieve highest nanobubble velocities and more dynamic beam steering. We expect that the higher the velocity of ONBs, the higher the probability and distance of ONB penetration into the tumor.

### Beam Steering of ONB in Bladder Cancer Mouse Model

**Figure 2:**
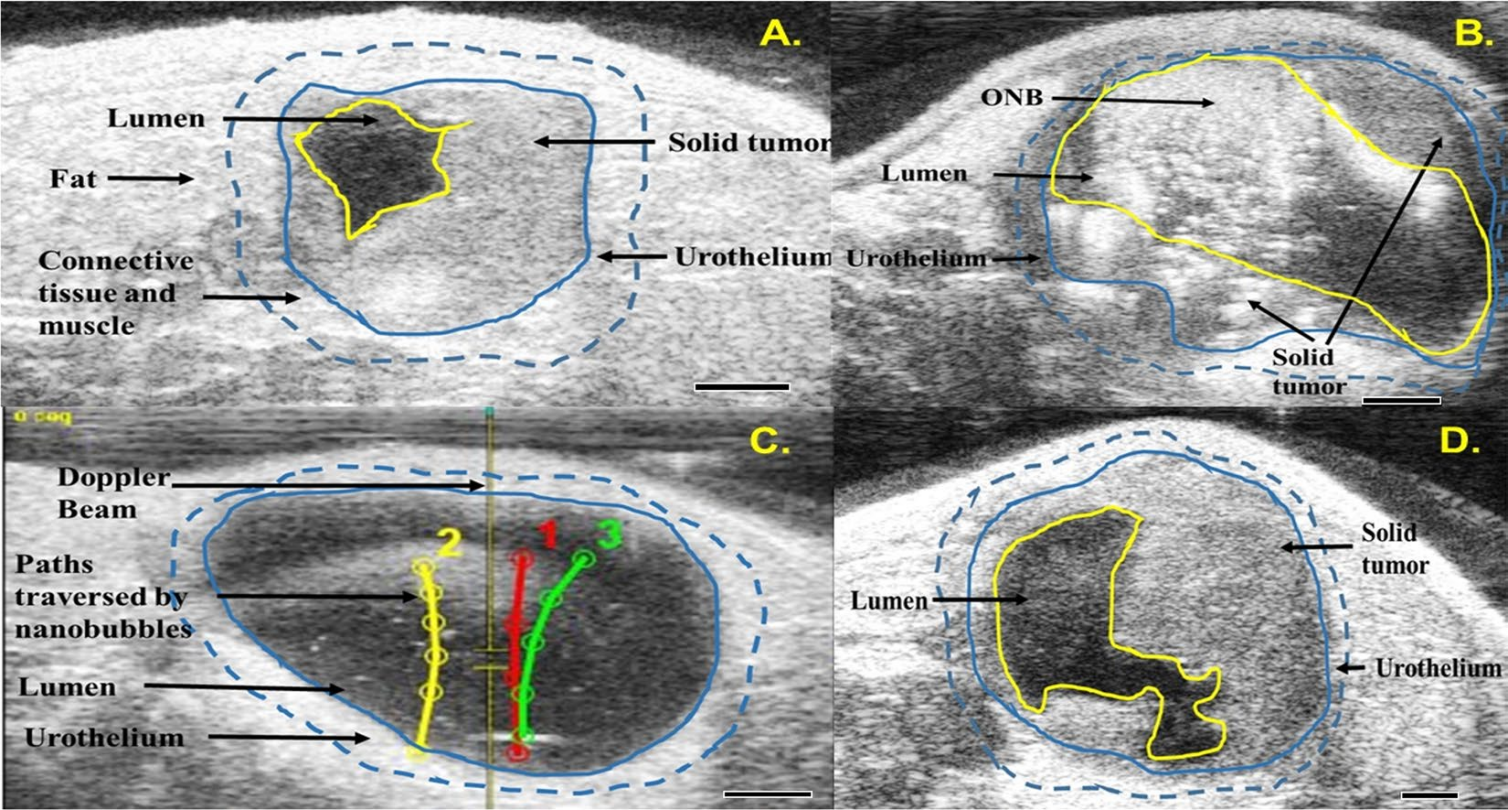
Precise steering of nanobubbles to the urothelium tumor after bladder instillation in mouse models. (**A**) B-mode ultrasound image of mouse bladder (dotted blue outline) showing tumor (blue outline) and bladder lumen (yellow outline). (**B**) Bladder injected with ONBs significantly enhance ultrasound contrast. (**C**) Nanobubbles are propelled (trajectories for selected three ONB streams are highlighted) along the DUB at 0° angle relative to the transducer. (**D**) Day 10 B-mode ultrasound image.

Next, the parameters established *in vitro* and *in vivo* were utilized to scale up the evaluation of oxygen nanobubbles (ONB) in mouse models and to explore a preliminary model for enhanced localization using beam steering. B-mode ultrasound clearly outlined the heterogeneous MB49 tumor implanted in the bladder wall (Fig. 2A). The treatment groups for our experiments were: (a) saline (**CTRL**); (b) mitomycin-C (**MMC**, 1 µg/mL), and (c) oxygen nanobubbles encapsulated with mitomycin-C (**ONB-MMC**, 0.5 µg/mL MMC and 2 mg/mL ONB). Based on our *in vitro* experiments, the concentration of MMC in ONB-MMC group was chosen to be 50% lower compared to the MMC group, since we hypothesized that beam steering can enhance the efficacy of the ONB-MMC group and results in significant tumor reduction, *in vivo*. Further, since our *in vitro* results indicated that beam steering was not significant in improving cell death in cells treated with ONB only, the ONB only group was not included in *in vivo* experiments.

**Figure 3:**
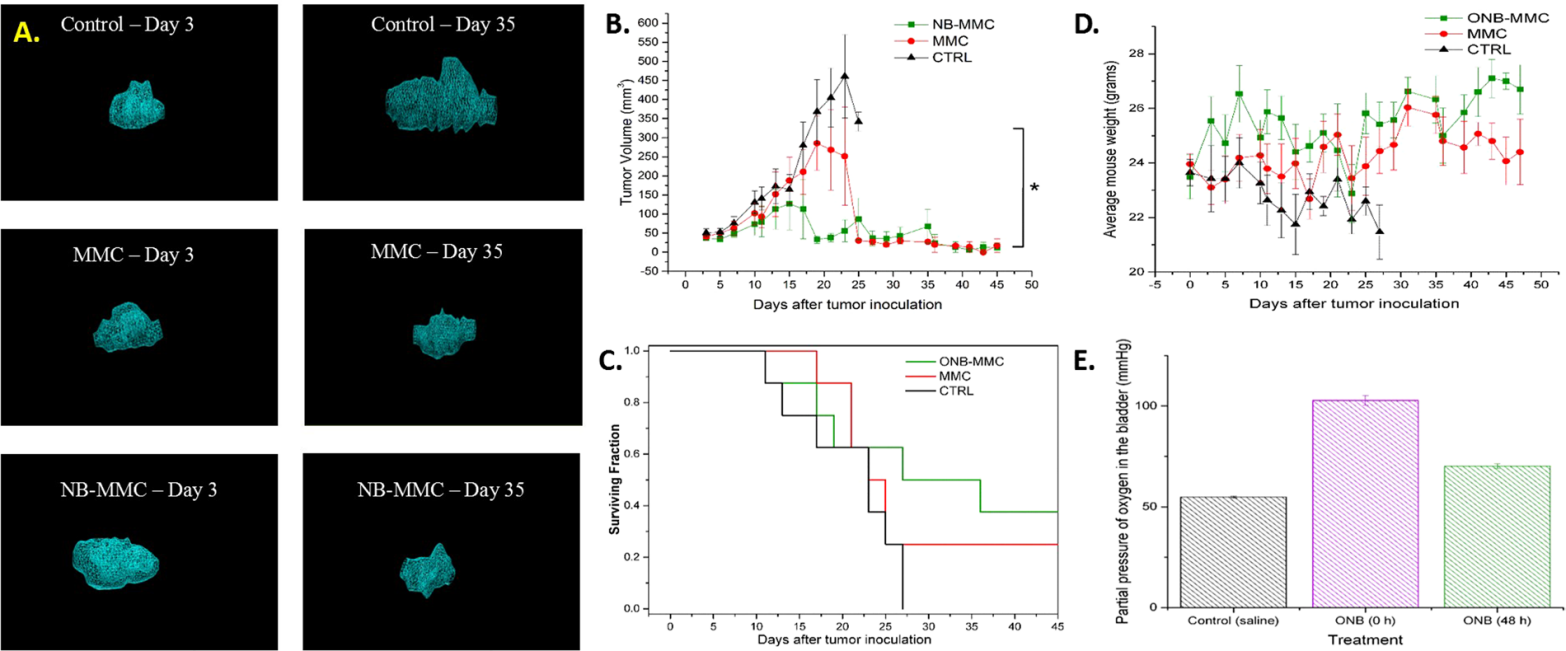
Ultrasound beam guiding of ONBs enhances therapeutic efficacy of MMC and reduces toxicity of treatment. (**A**) Reconstructed 3-D images of intravesical tumor for control (CTRL), MMC and ONB groups on day 3 and day 35. (**B**) Kaplan Meier survival curves for CTRL, MMC and ONB groups (n = 8 mice per group). (**C**) Tumor volume dynamics for CTRL, MMC and ONB groups (n = 8 mice per group). (**D**) Mice weight dynamics for CTRL, MMC and ONB groups (N = 8 mice per group) (**E**). Partial pressure of oxygen in the CTRL group, ONB group after 48 h of injections and ONB group after 0 h injection (n = 10).

Upon intravesical injection, the ONBs can be visualized in the bladder lumen (yellow highlight, Fig. 2B). Briefly, enhanced localization of beam steering was demonstrated *in vivo* in syngeneic orthotopic solid bladder tumor (Fig. 2C, Supplementary Video). Tumors were established in C57BL/6 J mice by intravesical instillation of MB49 cells. 2D (Fig. 2D) and 3D (Fig. 3A) volumetric quantification of the tumors and monitoring of tumor development was repeatedly and longitudinally evaluated in mice using the Vevo 2100 Ultrasound Imaging System (FujiFilm VisualSonics Inc., Toronto CA), equipped with segmentation tool for 3D images^30^. For intravesical therapy, the treatment groups (CTRL, MMC, ONB-MMC) were injected via a catheter in the mouse bladder every other day. Ultrasound Doppler Beam was then applied to each group every other day to induce beam steering and 0° beam angle was used to guide the nanobubbles. The nanobubbles were propelled along the beam and were precisely guided towards the tumor (Fig. 2C and Supplemental Video).

3D tumor reconstructions were performed for each representative mouse from ONB-MMC, MMC, and CTRL groups. A 3D ultrasound video of the tumor was broken down into individual 2D slices. The tumor contour was drawn for each 2D slice; all the 2D images were then lofted to create a 3D volume. 3D ultrasound image reconstruction from 2D images is not only an excellent visualization toolkit, but also offers accurate measurements of tumor volumes for comparison across ONB-MMC, MMC, and CTRL groups.

Figure 3A shows the reconstructed 3D volumes of representative mice from three study groups on day 3 and day 35. The visualized tumor volumes for CTRL group clearly shows a significant increase in tumor volume from day 3 to day 35. The 3D images of MMC tumor volumes are slightly different, with the tumor on day 35 being slightly smaller than on day 3. 3D reconstructions of ONB-MMC group tumors show a significant decrease in tumor volume from day 3 to day 35.

Further, we found that ONB-MMC group showed significantly slower tumor progression rate compared to the MMC-only group (Fig. 3B). Both, MMC and ONB-MMC groups were significant in reducing the tumor volume compared to CTRL. ONB-MMC also showed prolonged survival compared to MMC-only and CTRL groups (Fig. 3C). On day 22, there was a significant decrease in the surviving fraction of mice likely due to the toxicity of mitomycin-C (Fig. 3C). Hence, a significant drop in tumor volume for the MMC group of mice was observed (Fig. 3B). No adverse effects were observed in mice treated with ONB-MMC whereas two acute toxicity events were recorded in mice treated with MMC only which necessitated the mice to be euthanized. This shows that the ONB treatment reduces the toxicity of MMC. Further, the average mouse body weights were significantly lower for MMC and CTRL group mice compared to ONB-MMC groups indicating the reduced toxicity obtained because of the lower dose of MMC and reduction in systemic toxicity by ONB (Fig. 3D). Oxygen measurements were performed to evaluate the extent of oxygenation obtained in the mouse bladder. Oxygen content in the mouse bladder was measured in control mice and in ONB-MMC mice before and after the injection of ONB-MMC. Results show that ONB-MMC oxygen content before the injection of ONB is significantly higher than that of the CTRL group, indicating that ONB can provide a steady oxygen release and continuous oxygenation of the hypoxic regions of the tumor (Fig. 3E). These results demonstrate the ability of ONB in enhancing the performance of MMC, allowing the use of significantly lower concentration of chemotherapeutic drug, and prolonging survival rates in MB49 bladder cancer mice.

**Figure 4:**
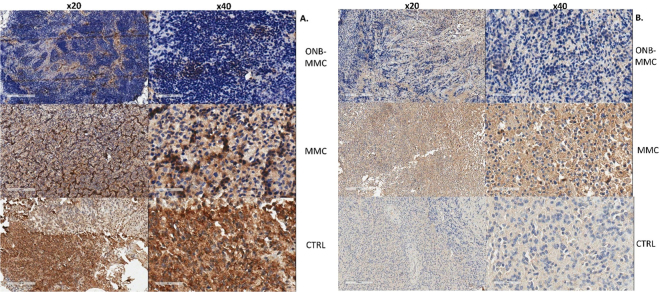
Immunohistochemistry staining pattern of hypoxic morphology markers. Microscopic images of mouse bladder with MB49 tumors treated with saline (CTRL), MMC only, or ONB-MMC (magnification left panel x20, scale bar = 200 µm; right panel x40, scale bar = 100 µm): (**A**) Strong HIF-1 expression is observed in control tumors and weak positive expression in ONB treated tumors (brown cytoplasmic staining). (**B**) Representative IHC images show that VEGF expression levels were significantly lower in ONB-MMC treated groups compared to CTRL or MMC groups (P  < 0.05).

We further characterized the reversal of chronic hypoxia due to oxygen nanobubbles in the *ex vivo* mouse bladder by quantifying and visualizing the expression of VEGF, HIF, and CD31 using established protocols^31–34^. We found that control tumors stained positive (brown coloration highlighted with arrows show positive cytoplasmic staining) for both, VEGF (Fig. 4A) and HIF-1 (Fig. 4B), consistent with previous reports in literature^6,34,35^, indicating that hypoxia is a critical stimulus for angiogenesis via up-regulation of the expression of VEGF and HIF. Angiogenesis and high density of blood vessels has been correlated with poor prognosis, metastasis, and bladder cancer invasion in clinical studies^6,36–38^. Compared to control, the VEGF and HIF-1 levels were significantly lower in bladder tissues of mice treated with ONB-MMC and MMC. The effect was more pronounced in ONB-MMC treated mice compared to only MMC since oxygenation because of ONB is probably due to significant reversal of hypoxia in the tissue. The significantly lower expression of HIF and VEGF in ONB-MMC samples indicates that the lower tumor volumes observed could be due to anti-angiogenesis and hypoxia targeting potential of ONBs. Overall, the different staining presented indicate the different anti-angiogenesis mechanisms triggered because of the reversal of hypoxia obtained using ONB.

### Validation of ONB Localization using Hyperspectral Dark-Field Microscopy

**Figure 5:**
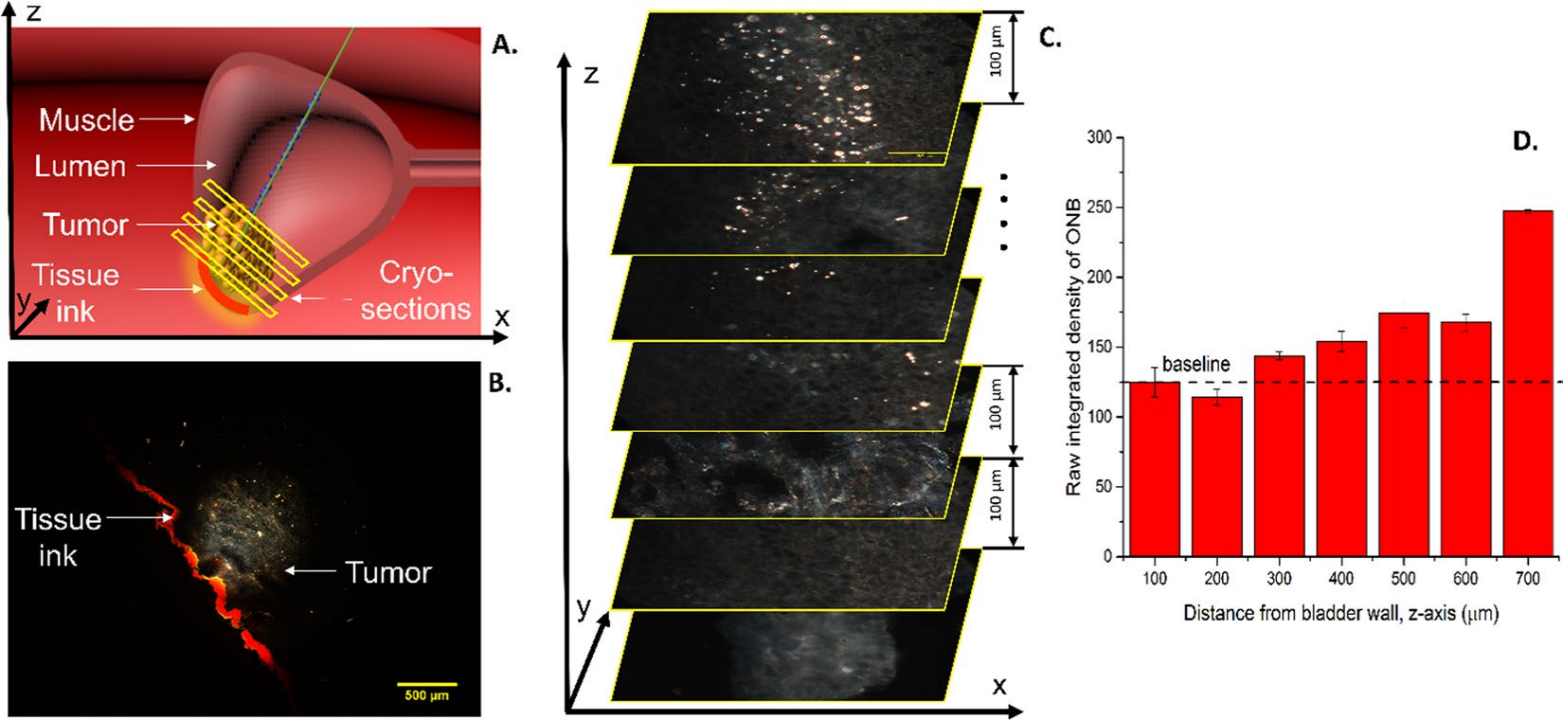
Nanobubbles can be localized up to 500 µm inside the tumor using beam steering. (**A**) Schematic of bladder tumor stained with tissue ink during dissection to maintain orientation and cryosectioned using a cryotome. (**B**) Dark-field image showing tissue ink used for maintaining sample orientation and bladder tumor. Image obtained at x10 magnification. Scale bar = 500 µm. (**C**) Representative dark-field images showing localization of ONBs (bright spots) up to a depth of 500 µm inside the tumor. Cryosections were obtained at intervals of 100 µm and thickness of tissue sections was 10 µm. Bottom image (z-axis) represents bladder wall whereas top-most image represents the outer layer of tumor wherein ONBs penetrate using the Doppler beam. (**D**) Raw integrated density of ONBs shows significant accumulation of ONBs up to 300 µm from the bladder wall (500 µm from tumor periphery). Baseline represents background signal obtained from CTRL mouse bladder. The amount of ONBs decreases with distance from the tumor periphery.

To confirm the localization of ONBs inside tumors due to beam steering, dark-field images of cryosectioned tumor tissues were obtained and the ONBs at successive layers were quantified using ImageJ^39^. First, the mouse bladder was stained with tissue marker dye to maintain orientation during dissection post-euthanasia (Fig. 5A). Further, the tissues were cryopreserved and sectioned using a cryotome. Successive sections were obtained every 100 µm from the bladder wall. Next, the tissue sections were imaged with a dark-field microscope using a previously published protocol^29^. The bladder orientation was maintained using the tissue ink applied during dissection that was observed at x10 magnification (Fig. 5B). We found that ONBs were clearly visible up to 500 µm sections from the tumor periphery (Fig. 5C, **top image**) confirming the presence of nanobubbles inside the tumor tissue. The amount of nanobubbles decreased with distance from the tumor periphery indicating that the Doppler penetration had limitations on the distance of penetration (Fig. 5D). The mechanical force of the ultrasound Doppler beam can be tuned by changing beam power, frequency, and time of insonation to obtain the desired depth due to steering of nanobubbles.

### Prediction of ONB Velocity using Modeling

**Figure 6:**
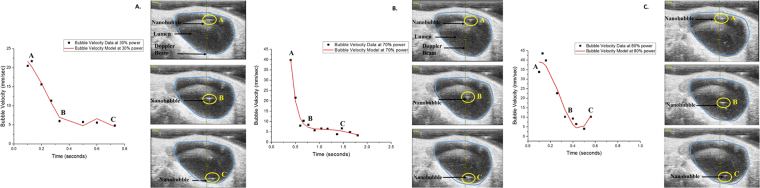
Nanobubbles can be propelled at desired velocities by tuning the ultrasound Doppler beam power as predicted by the theoretical model. Nanobubble velocity data (black squares) and model (red line) for 30% (**A**), 70% (**B**), and 80% (**C**) ultrasound beam power. Points A, B, and C indicates the bubble’s position at a given time.

A theoretical model was developed to predict the velocity profile of nanobubbles through the media (urine inside the bladder) and to comprehensively approximate the pressure profile inside the bladder and, finally, outline the significant forces acting on the bubble (Fig. 6, Supplementary Information). The forces taken into account were Bjerkens force (results from out of phase bubble oscillations and ultrasound wave oscillations)^40^, buoyancy force (the weight of the liquid displaced by the bubble)^41,42^, and drag force (force, acting opposite to the direction of the bubble movement in the liquid)^43^. Gravitational force was neglected due to the insignificant mass of ONB compared to other contributing forces. It is known that ultrasound pressure wave can be represented as a harmonic plane wave. Bjerkens force is acting downward on the bubble, and is represented by the product of the volume of the bubble and the gradient of the pressure wave. The average Reynold’s number was calculated as 3.75 × 10^−3^, indicating a laminar flow and linear dependence of Drag force on the bubble velocity. The objective was to derive the velocity profile of ONB as it is propelled by the transducted pressure wave in the lumen. The defining differential of Newton’s law was coupled with the expression of the net force acting on the bubble and was further integrated with respect to time to yield the velocity profile presented in equation (1).

1 $$v(t)=A\cdot \cos (\omega t)-B\cdot \sin (\omega t)+C\cdot {e}^{-\alpha t}$$

where A, B, C, and $\alpha$ are constants that depend on the mass, volume and radius of the bubble, urine viscosity and density and gravitational acceleration. *ω* is the angular frequency of the pressure wave. The equations for the coefficients and a detailed explanation of the model can be found in the Supplemental Information.

Figure 6 shows the velocity data and a theoretical model for the ONBs’ ultrasound propelled motion. The obtained model shows the nanobubble velocity profile as it moves from the top to the bottom of the bladder (bladders with no tumor were used for data collection). As the bubble moves further away from the transducer and the intensity of the pressure wave deteriorates, the ONB velocity progressively decreases. It can also be noticed that the maximum velocity increases as the ultrasound power is increased from 30% (Fig. 6A) to 70% (Fig. 6B). It should be noted that the intravesical treatments and beam steering were performed at 100% power (Fig. 6C), where the nanobubble velocity is highest. Further, since the traveling distance to the tumor is much shorter than the distance travelled in an empty bladder, the velocity of bubble penetration in the tissue would be much higher that the terminal velocity of the nanobubbles in the empty bladder (Fig. 6, **point C**).

Overall, we have demonstrated enhanced efficacy and reduced toxicity of MMC using ONB to revert hypoxia. We show that ONB-MMC were precisely guided and powered into the tumor with ultrasound image-based Doppler beam steering. We have optimized the velocity, magnitude of accumulation, and distance traversed in response to ultrasound frequency, power, and beam angle to effect ONB guided localization. Finally, the changes in hypoxia-adaptive pathways in the orthotopic mouse bladder tumors because of MMC and ONB were studied.

## Discussion

We have demonstrated that oxygen nanobubble carrier for mitomycin-C can be precisely guided to hypoxic bladder tumors and can enhance the effectiveness and reduce the toxicity of conventional chemotherapy. The ONBs were guided using an ultrasound Doppler beam to the tumor and the acoustic ‘bombardment’ of the nanoparticles enhances its uptake by the tumor cells. This new concept was illustrated using MB49 bladder tumors syngenically grown in mouse bladders. Efficient steering of the nanobubbles was demonstrated with a velocity of over 30 mm/s in the mouse bladder. The mitomycin-C dose was reduced by half to attain a similar efficacy in therapy. Further, ultrasound Doppler beam parameters such as angle, power, and frequency and their effects on the nanobubble velocity and acceleration were investigated using a theoretical model.

## Conclusion

In conclusion, we have shown that oxygen trapped nanobubbles could be guided by ultrasound to enhance the efficacy of localization and targeting for reverting hypoxia in NMIBC tumors. We have demonstrated image guided enhanced localization (~100 µm resolution) of particles with Doppler ultrasound beam to a desired location both *in vitro* and *in vivo* with ultrasound steering. Our experiments and calculations showed that the nanobubbles could be propelled to a velocity of ~40 mm/sec at various angles (−15°, 0°,15° beam angle) with ultrasound steering. Our results show that nanobubbles were found to localize 500 µm deep into the bladder wall tumor from the periphery using beam steering inside the mouse bladder. The ability to precisely steer nanobubbles loaded with a payload using non-invasive and safe image-guided ultrasound offers considerable promise for designing targeted cargo delivery and hypoxia reprogramming for a wide range of biomedical applications.

## Materials and Methods

### Cell culture

The mouse urothelial carcinoma cell line MB49 was used for *in vitro* and *in vivo* experiments because it is a widely used and well-studied mouse bladder cancer model. Cells were cultured in DMEM/F-12 media with addition of 10% Fetal Bovine Serum and 1% Penicillin (10,000 I.U/ml) – Streptomycin (10,000 µg/ml). The cells were cultured at 37 °C in a humidified atmosphere with 5% CO_2._ Cells were tested for mycoplasma contamination using Hoechst 33258 fluorescent indirect staining^44^ before initiating the experiments. Briefly, cells were fixed using 4% paraformaldehyde (PFA) solution and stained with Hoechst 33258 fluorescent dye. Images were obtained using a confocal microscope. No small specks were observed surrounding the cells thus confirming the absence of mycoplasma.

### Intravesical tumor instillation

All experiments were performed in accordance with relevant guidelines and regulations per our protocol approved by Purdue Animal Care and Use (PACUC) committee (approval # 1404001052). Study was performed with blinding for all researchers collecting data except the first author PB for whom blinding was not possible. Female C57BL/6 mice were anesthetized by 1–2% isoflurane gas inhalation at 2 L/min. Electrocauterization technique was used to create a minor injury on the wall of the bladder for MB49 cells to adhere to. A 24 gauge Teflon catheter (Terumo Surflo PTFE I.V. Catheters, Fisher Scientific) was inserted into the bladder through the urethra. Minor pressure was applied onto the bladder area to release the urine from the bladder. A wire, connected to the electrocautery unit was then instilled into the bladder through the catheter. 1 × 10^4^ MB49 cells were then instilled into the bladder; dwelling time was approximately 20 minutes to allow the cells to adhere to the injured bladder wall.

### Synthesis of oxygen nanobubbles and mitomycin encapsulation

ONBs were synthesized by modifying our previously published protocol^27,29^. Briefly, sodium carboxymethyl cellulose (NaCMC) hydrogel (FMC Biopolymer) an FDA-approved excipient was cross-linked while encapsulating the oxygen inside the gel using a layer-by-layer (LBL) approach^45^. NaCMC (Ac-Di-Sol, FMC Biopolymer, Philadelphia, PA) was dissolved in nanopure water to form a 0.05% (w/v) gel and homogenized and saturated with gaseous oxygen (UHP grade) with a nanosize air nozzle (EXAIR Corporation) and a 20 nm filter (Emflon II, Pall Corporation) to help generate oxygen NBs. After sonication^46^, 0.05% aluminum chloride (AlCl_3_), a trivalent cross-linking agent which provides exquisite stability^47^, was added to form the encapsulation under continuous ultrasonication and adjusted to a neutral pH. To synthesize ONB-MMC, required amounts of mitomycin-C (LKT Laboratories) was dissolved in nanopure water before initiating the ONB synthesis. The concentration and encapsulation efficiency of MMC was measured spectrophotometrically at 364 nm using a NanoDrop UV-Vis spectrophotometer (ND-1000, ThermoFisher Scientific)^48^.

### Cell count and cell viability

Cell count and cell viability measurements were performed before and after the experimental procedure for initiation and completion respectively. To approximate the number of live and dead cells in the sample the Countess® Automated cell counter (Invitrogen, Life Technologies) was used. The original sample dilution was carried out in 0.4% trypan blue (Sigma Aldrich) and further diluted by a factor of 2. The prepared samples were deposited onto the punter slides and used for cell counting.

### *In vivo* ultrasound imaging and beam steering

Vevo 2100 ultrasound imaging system (Fujifilm VisualSonics Inc., Toronto CA) equipped with a 22–55 MHz microscan transducer (MS550D, Vevo 2100, VisualSonics Inc.) with a center frequency of 40 MHz. Imaging focal zones, brightness, and contrast were kept constant throughout the experiments. Mice were anesthetized using 1–2% isoflurane in 2 L/min of medical air and restrained on a heated platform. B-mode and 3D image were collected every other day for each mouse.

After the bubbles were injected intravesically, a pulsed wave Doppler beam was applied for five minutes and B-mode images were recorded. 100% percent power was consistently used, since it was shown to result in the highest average nanobubble velocity. The mouse urethra was taped and closed for 1 hour per previously published protocols^48,49^. Closing the urethra ensures that the chemotherapeutic does not leak out immediately with urine from the mouse bladder.

### ONB-MMC and MMC treatment regimens

Mice were randomized into three groups: ONB-MMC (Mitomycin-C encapsulated in the oxygen nanobubble), MMC only, and saline (CTRL) (N = 8). The mice were imaged 48 hours after the instillation of the tumors and treatment was administered every other day based on previously established protocols^49–51^. ONB-MMC, MMC only, and saline (CTRL) group mice then received 100 µL injections with optimized concentrations, every other day.

### Tumor volume analysis

3D images^52^ of bladder tumor were taken every other day using the Vevo 2100 ultrasound imaging system (Fujifilm VisualSonics Inc., Toronto CA). Vevo Lab software was used to manually segment given 3D images in 2D slices, with step sizes that can be as small as 10 µm. Segmentation of a 3D image allowed accurate volume measurements to be taken from each 3D image. Manual segmentation was used to outline the tumor for each 2D image segment, which were then lofted together to reconstruct the tumor and estimate its volume.

### Oxygen release measurement

Oxygen content in the mouse bladder was measured using OxyLite oxygen probe (Oxford Optronix Ltd). The SURFLO® ETFE I.V. 24 Gauge Teflon Catheter was instilled into the bladder and slight pressure was applied on the bladder to release the urine. Oxygen probe was then instilled into the bladder through the catheter and 10 measurements of oxygen partial pressure were recorded.

### Histology analysis

Histology slides were prepared and immunohistochemistry was performed at Immunohistochemistry Laboratory at IU Health Pathology. Harvested organ tissue and bladder tumors were fixed in formalin-free IHC Zinc Fixative, embedded in paraffin, and sectioned into 5 µm slices. HIF-1 and VEGF immunohistochemistry was performed using buffered zinc formalin fixed tumor section. Blocking, antigen retrieval, and primary and secondary antibody staining were performed simultaneously. The tissue sections were imaged using Leica DMi1 inverted microscope, and image processing was performed using ImageJ software (Research Services, National Institute of Health). Each tissue section was imaged under x20 and x40 magnification, composite images were converted to RGB stacks and the integrated density of staining was calculated.

### Cryosectioning

Mouse bladders were cryosectioned prior to dark-field imaging using a published protocol^53^. First, the mouse bladder was dissected immediately after euthanizing the mouse. The entire bladder was frozen in a cryomold cassette (Fisher Scientific, NC9969692) with OCT compound (Fisher Scientific, 23-730-571). The bladder orientation was maintained by staining the bladder with tissue ink (Davidson Marking System, Bradley Products, #1101). Further, bladder tissue sections of 10 µm thickness were cut using a cryotome (Cryotome FE, ThermoFisher Sci.) with a spacing of 100 µm between every section. The tissue sections were imaged using dark-field microscopy.

### Dark-field microscopy

Dark-field imaging of the cryosectioned tissues were performed using a previously published protocol^29^. Briefly, a home-built hyperspectral dark-field microscope (HSDFM) was used for imaging the tissue sections^54–56^. The samples were illuminated using a tungsten halogen source (3900, Illumination technologies Inc.) and a CytoViva dark-field condenser (NA 1.2–1.4) with a fiber optic light guide. The scattering light was collected with either a 10x or 100x oil immersion objective. The tissue orientation was maintained by observing the samples first at low magnification x10 and identifying the tissue ink on the bladder wall. Once the tumor is located, the x100 magnification can be used to obtain hyperspectral images.

### Quantification of ONB in HSDFM images

The ONBs in the HSDFM images were quantified with ImageJ software (NIH) per our previously published protocol^29^. The maxima function on ImageJ was used to obtain the raw integrated density of ONBs per area. The baseline was obtained by quantifying the signal in mouse tissue treated with saline (CTRL).

### Statistical data analysis

Statistical data analysis was performed using JMP Statistical Software (SAS Institute Inc., Cary, NC). Sample size was calculated using power law analysis. Assumptions for the tests were checked using normal probability plots and residual plot analysis in JMP software. Two sampled student’s unpaired t-test and Fisher’s least significant difference (LSD) test were used to determine the level of significance at 5% unless mentioned otherwise.

### Associated Content

Contour plot for cell viability (%) with beam steering (top) and without steering (bottom). Factorial design to optimize in vitro parameters for MB49 cell viability; Results of factorial design of experiments to identify significant parameters influencing nanobubble velocity; ONB velocity increases with increase in ultrasound beam power; Theoretical model to predict the behavior of nanobubbles and the fundamental mechanisms governing the beam steering (PDF).Ultrasound beam steering of oxygen nanobubbles *in vitro* and *in vivo* (AVI).

## Electronic supplementary material

Supplementary video

Supporting Information
