# Supplementary material for: Ultrasound beam steering of oxygen nanobubbles for enhanced bladder cancer therapy
Source: Sci Rep. 2018 Feb 15;8:3112. doi: 10.1038/s41598-018-20363-8 (PMC5814559; doi:10.1038/s41598-018-20363-8)
Supplement: Supplementary file 2 — Supporting Information [file 41598_2018_20363_MOESM2_ESM.pdf]

Supporting Information for

Ultrasound beam steering of oxygen nanobubbles for enhanced bladder cancer therapy

Pushpak Bhandari,[1,4] Gloriia Novikova,[2,4] Craig J. Goergen,[3,4] and Joseph Irudayaraj[1,4,]*

[1]Department of Agricultural and Biological Engineering, Bindley Bioscience Center, Purdue University, West Lafayette, Indiana 47907, United States

[2]Davidson School of Chemical Engineering, Purdue University, West Lafayette, Indiana 47907, United States

[3]Weldon School of Biomedical Engineering, Purdue University, West Lafayette, Indiana 47907, United States

[4]Purdue University Center for Cancer Research, West Lafayette, Indiana 47907, United States

Supplementary Methods and Materials

Contour Plot for Cell viability (%) with Beam steering=OFF

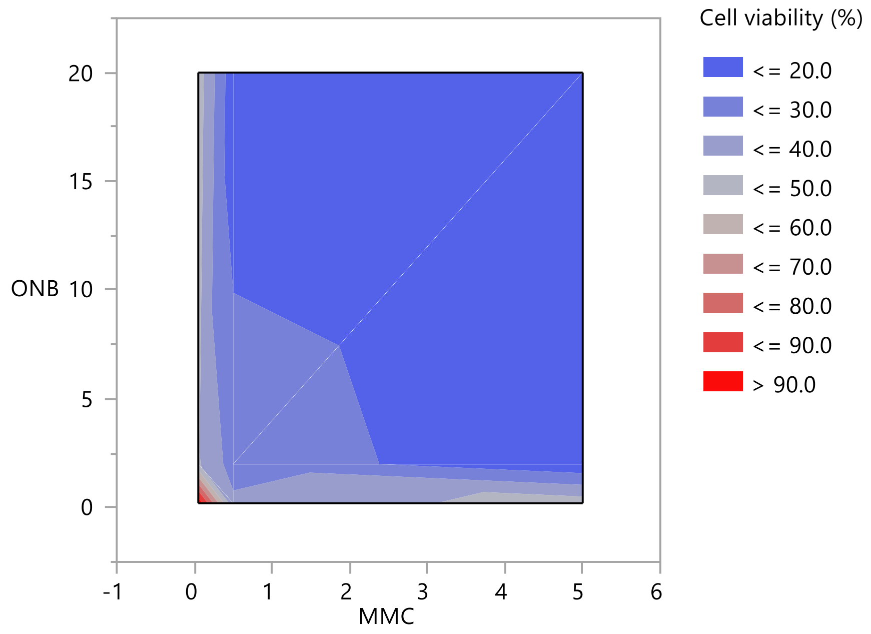

Contour Plot for Cell viability (%) with Beam steering=ON

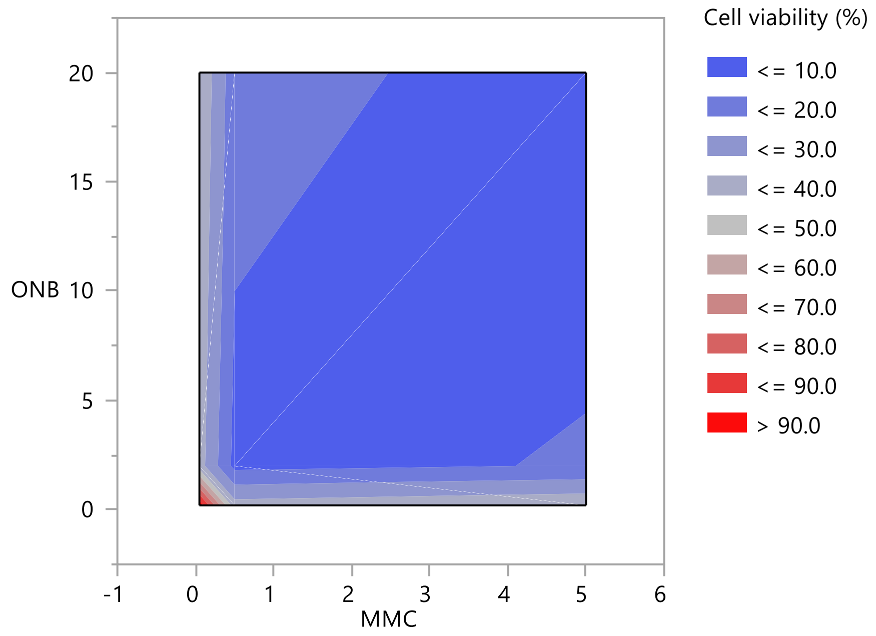

Fig. S1: Contour plot for cell viability (%) w/o beam steering (top and bottom respectively). Ultrasound Doppler beam significantly reduced cell viability when ONB-MMC were both at medium concentrations.

Table S1: Factorial design to optimize *in vitro* parameters for MB49 cell viability.

Parameter	Range		Results
MMC	0.05 µg/mL	Low	Significant
	0.5 µg/mL	Medium	
	5 µg/mL	High	
ONB	0.2 mg/mL	Low	Significant
	2 mg/mL	Medium	
	20 mg/mL	High	
Beam steering	OFF	Low	Significant when ONB-MMC are both at medium concentration
	ON	High	
Cell Viability (Y)	0-100 %		

Table S2: Results of factorial design of experiments to identify significant parameters influencing nanobubble velocity. Utrasound beam frequency and power were significant in changing the velocity of ONB.

Parameter	Estimate	F ratio	Prob > F
Intercept	26.71	0.00	1
ONB Size	0	2.93	0.16
Beam frequency	-1.03	45.69	0.001
Beam power	0.654	18.45	0.007
Beam angle	0	0.35	0.587

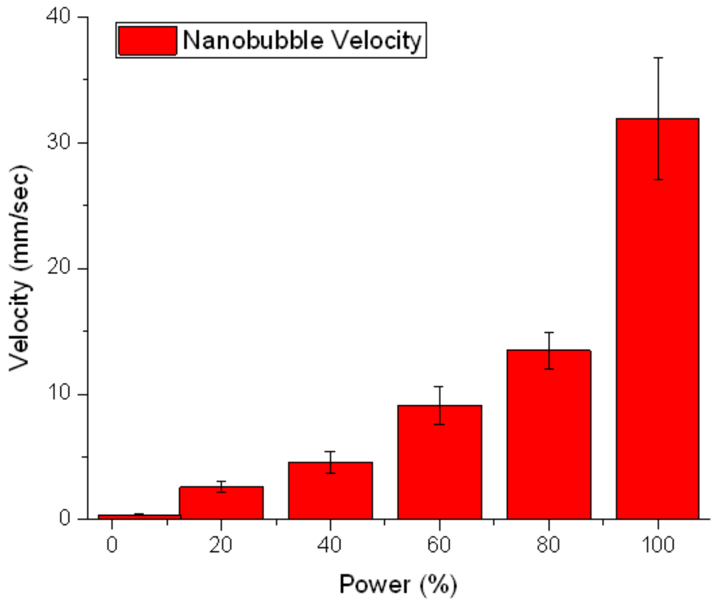

Figure S2: ONB velocity increases with increase in ultrasound beam power.

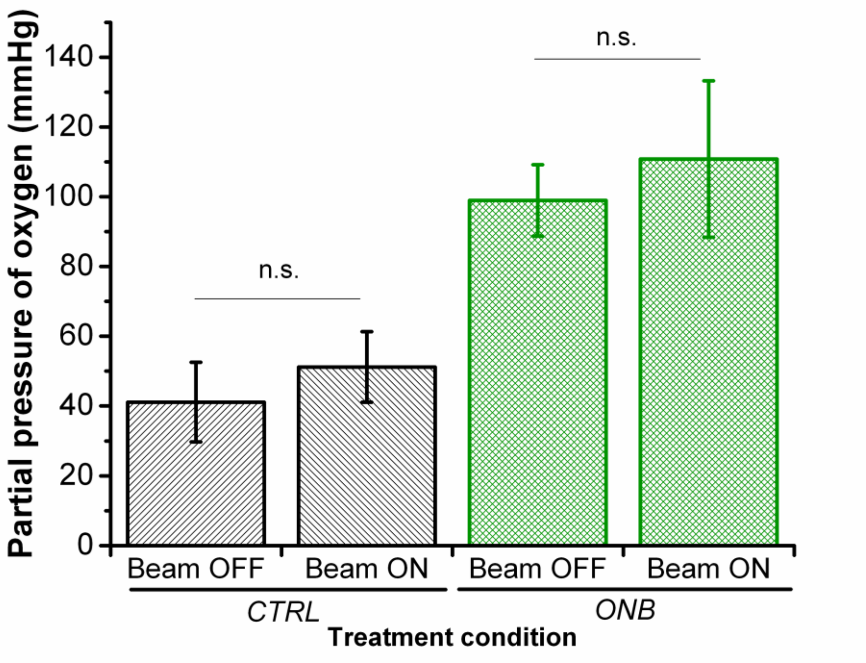

Figure S3: Effect of beam steering on the partial pressure of oxygen for CTRL (saline) and ONB treated MB49 cells *in vitro*. MB49 cells were incubated with CTRL (saline) or ONB (2 mg/mL) for a period of 24 h. Partial pressure of oxygen was measured with an OxyLite probe inserted aseptically into the cell culture. pO2 readings are averaged over recordings made every 60 s for 5 min. (n = 3).

Method S1. Theoretical model to predict the behavior of nanobubbles and fundamental mechanisms governing beam steering.

A theoretical model to predict the velocity ONBs was developed, taking into account the nature of the media (urine inside the bladder), comprehensively approximating the pressure profile inside the bladder and, finally, incorporating the significant forces acting on the bubble. The forces considered in this model are Bjerkens force, buoyance force, drag force and gravitational force. The primary Bjerkens is responsible for the translational motion of the bubble and results from out of phase bubble oscillations and ultrasound wave oscillations. Buoyancy force, known as the force exerted by the fluid on an object immersed in it, equals the weight of the liquid displaced by the bubble. Drag force is acting opposite to the direction of the bubble movement in the fluid. Gravitational force acting on the nanobubble is neglected due to the infinitesimal mass of the bubble.

Bjerkens Force.

The frequency of the wave, the base pressure (amplitude) corresponding to the transducer used are known and are constant. The distance between the transducer and the bubble can be easily approximated. An accepted standard in the field of acoustics is an assumption that the pressure wave is made of small amplitude oscillations and is written as following:

$$P(z,t) = P_A \cdot \cos(\omega t - kz) \tag{1}$$

By inspecting equation (1), relative magnitudes of the terms wt and kz were estimated. Since k = $\frac{w}{c}$, where c is the speed of light in urine (for reference, the speed of sound in water is 1484 m/s). z, which represents the distance along the bladder, has a magnitude of 10^{-3} m. It becomes clear that the magnitude of wt is significantly larger than that of kz; thus, kz can be omitted from the equation to simplify initial modeling.

The wave equation thus becomes the following:

$$P(t) = P_A \cdot \cos(\omega t) \tag{2}$$

Since the volume of the bubble was assumed to be constant (oscillations are negligible, bubble mean radius is a considerably smaller than the wavenumber), mean Bjerkens force is as follows:

$$F_{Bjrk} = [V(t)\nabla P(z,t)] \tag{3}$$

Since the volume of the bubble is assumed to be constant, the average Bjerkens force acting on the bubble can be written as:

$$F_{Bjrk} = -V \cdot \omega \cdot \sin(\omega t) \tag{4}$$

Buoyancy Force.

The buoyancy force is constant, and can be expressed as follows:

$$F_B = \rho_{fluid} \cdot (\frac{4}{3} \cdot \pi \cdot R^3) \cdot g \tag{5}$$

Drag Force.

Average velocity of the bubble was used to calculate Reynold number to characterize the type of flow. The average velocity was found to be 6.6 mm/s at 40% power and 22 mm/s at 100 % power. Thus, the Reynolds number was computed as follows:

$$\mathrm{Re} = \frac{\rho_{fluid} \cdot v \cdot D}{\mu} \tag{6}$$

Where ‘ρ’ is density of urine, ‘v’ is velocity of the nanobubble, ‘D’ is nanobubble diameter, and μ is the dynamic viscosity of urine. This calculation yielded a Reynolds number of 3.75×10^{-3}.

Since laminar flow exists when Re < 0.1, the drag force can be estimated as follows:

$$F_{drag} = b \cdot v \tag{7}$$

where,

$$b = 6\pi\eta R$$

‘η’ is the urine viscosity and ‘R’ is radius of nanobubble.

The resulting net force can thus be expressed as following:

$$F_{net} = F_{Bjrk} - F_{drag} - F_{buoy} = -V \cdot \omega \cdot \sin(\omega t) - b \cdot v - \rho_{fluid} \cdot (\frac{4}{3} \cdot \pi \cdot R^3) \cdot g \tag{8}$$

Since the objective of the model is to describe the velocity profile of the bubble, the defining differential equation of Newton’s law was used and the net force acting on the bubble was plugged into the equation.

$$F_{net} = m \cdot \frac{dv}{dt} \tag{9}$$

$$F_{net} = F_{Bjrk} - F_{drag} - F_{buoy} = -V \cdot \omega \cdot \sin(\omega t) - b \cdot v - \rho_{fluid} \cdot (\frac{4}{3} \cdot \pi \cdot R^3) \cdot g = m \cdot \frac{dv}{dt} \tag{10}$$

Integration yields:

$$v(t) = -\frac{gm^2\rho V\omega^2}{b^3 + bm^2\omega^2} + \frac{bmV\omega^2\cos(\omega t)}{b^3 + bm^2\omega^2} - \frac{b^2 g\rho V}{b^3 + bm^2\omega^2} - \frac{b^2 V\omega\sin(\omega t)}{b^3 + bm^2\omega^2} + c_1 \cdot e^{-\frac{bt}{m}} \tag{11}$$

By analyzing the order of magnitude of each component of the solution, it was found that the constant terms are negligible since the ‘Vm^2’ term is on the order of 10^{-40} and Vb^2 term is in the order of 10^{-30}. Thus, we can ignore the constant. The simplified model is as follows:

$$v(t) = \frac{bmV\omega^2 \cos(\omega t)}{b^3 + bm^2\omega^2} - \frac{b^2V\omega^2 \sin(\omega t)}{b^3 + bm^2\omega^2} + c_1 \cdot e^{-\frac{bt}{m}} \quad (12)$$

The term preceding cosine is defined as '***a'***, the term preceding sine is defined as '***b',*** and the power of the exponential is defined as '***d'.***

The experimental data for 30% and 80% power were fit to the model and the results are shown in **Figure 6**.
